# Seamless phase IIa/IIb adaptive design with the same primary endpoint for proof of concept and dose finding

**DOI:** 10.1016/j.conctc.2018.06.006

**Published:** 2018-06-18

**Authors:** Jiacheng Yuan, Daniel Radecki, Denise Bugarin, Till Geib, Jihao Zhou, Jeen Liu

**Affiliations:** Allergan Inc., 2525 Dupont Drive, Irvine, CA 92612, USA

**Keywords:** Adaptive design, Dose finding, Proof of concept, Seamless design, Budget saving

## Abstract

This paper considers combining a proof of concept (POC) study and a dose finding (DF) study where the POC and the DF share the same primary endpoint. An example based on real study conditions shows that compared to a conventional design the proposed adaptive design tests more active doses, with a smaller sample size and a shorter overall duration leading to a budget saving of 30% in study operations.

## 1 Introduction

This research was motivated by the trial design of a real Allergan project. The immediate release formulation (IRF) of a compound has been sufficiently studied at Allergan. There has been a new delayed release formulation (DRF), on which the adaptive design that we discuss here is to apply. The DRF showed good preclinical evidence for an impact on animal models of visceral pain. Clinical studies of good sample size (over 60 patients per dose level) have already been conducted in a visceral pain population utilizing this DRF. The clinical data demonstrate no dose response over a 10-fold range, which means either there is no efficacy or the studied doses are on the flat part of the response curve. Based on the preclinical data, it is plausible to assume that this range is on the lower flat part of the response curve. Under this situation, it is necessary to study a wide range of doses to get to see efficacy. With high level doses, the AE of interest manifests quickly based on the phase I study with the IRF.

In a conventional approach, after the maximum tolerated dose has been identified in phase I studies of healthy volunteers, a POC study may be performed with a high dose versus placebo, and afterwards a DF study may be performed with three doses versus placebo. For this project, POC and DF have the same primary endpoint, Week 12 maximum pain. Based on results from previous controlled trials in this population, we assume the treatment difference (active treatment - placebo) in Week 12 maximum pain is −1.3 with a common standard deviation of 2.53. Let the POC study have 48 patients per arm, then the power is 70% with a two-sided 0.05 alpha. Let the DF study have 60 patients per arm, then the power is 80% with a two-sided 0.05 alpha, without adjustment of multiplicity. This results in a total of 336 patients to evidence potential effectiveness and identify dose levels for phase III program.

An adaptive design proposed by the authors requires fewer patients but assesses more doses and finishes in a shorter duration. The rest of the article is organized as follows. Section 2 summarizes a previous related design from Ref. [2]. The seamless phase IIa/IIb design with the same primary endpoint in the two stages is elaborated in Section 3. The proposed adaptive design is compared to the conventional design on statistical operating characteristics as well as clinical operations in Section 4. The paper concludes in Section 5 with some discussions.

## 2 Seamless phase IIa/IIb design with different primary endpoint in two stages

[2] introduced an adaptive design that combines a proof of concept (POC), usually called phase IIa, study and a dose-finding (DF), usually called phase IIb, study. Its key features are to start with more dose levels, e.g. 6 or 7 doses, and conduct the trial in two stages, where in the first stage establish proof of efficacy via comparison of active drug to placebo using pooled groups excluding the worst performers, then recycle the majority of the placebo-treated patients into the second stage. In the second stage of dose finding, drop less promising doses sequentially, and in the end test superiority between the remaining individual active dose(s) and placebo. The primary endpoint assessed by the POC and the DF stages are similar but different, e.g. a biomarker versus a clinical endpoint or the same study endpoint evaluated at different timepoints. In this case the application of the seamless design demands much pre-planning and only fits for a limited number of indications which have relative infrequent dosing (e.g. once per month or once every two months), because it involves quick decisions whether to remain on placebo (to reach the DF primary endpoint time point) or switch to active treatments for patients started with placebo at the POC primary endpoint time point.

## 3 Seamless phase IIa/IIb design with same primary endpoint in two stages

This paper considers the situation where the POC stage and DF stage share the same primary endpoint, i.e. the same study endpoint with the same assessment period (AP). This simplifies the study operation, because it does not require a quick decision on which patient groups are better performers in the POC stage, even though the statistical analysis involves discarding data from some patient groups who have relative poorer performance. This simplification of the study operation makes it suitable for much more applications. The methodology is illustrated with a numerical example that starts with 6 active doses, drops 4 of them in two interim analyses, and finally conducts dose finding test with the best 2 versus placebo.

In terms of study operation, patients enrolled at the POC stage are in the study for a duration of 2*AP. Patients are randomized into the POC per treatment sequences. There are 2 sequences for every dose: one sequence called the immediate-treated subgroup in which patients are treated with an active dose in both AP's; and the other sequence called the delayed-treated subgroup in which patients are treated with placebo in the first AP and an active dose in the second AP. The two sequences of the same dose form a dose group in the POC. Please note the baseline is defined as the date of start of each treatment. Therefore, for patients in a delayed-treated subgroup the primary endpoint will be measured twice, at AP1 for placebo and AP2 for the subsequent active dose. The randomization is 1:1 between the two sequences of a dose, and all doses are balanced in the POC enrollment. There may be a second enrollment, the DF enrollment, which does not start until the POC succeeds and the DF doses have been decided with the data from the POC stage.

The primary endpoint is denoted as E, which is the same for the two stages and assumed to be continuous. Without loss of generality, a larger value in E represents a better condition. We adopt similar notations to [2]. At the end of the seamless study, a maximum of G patients can be enrolled to a dose of the active treatment, and G is the per arm sample size that is adopted by a conventional DF study. The POC stage enrollment starts with m active dose levels. Practically, m can be 6 or 7. For each dose, the corresponding group has two subgroups, immediate-treated and delayed-treated, where the half in the immediate-treated subgroup are dosed with active treatment at the beginning, while the other half in the delayed-treated subgroup are dosed with placebo at the beginning.

The trial is conducted in two stages, where in the first stage proof of efficacy is established via a comparison of active drug to placebo using pooled groups excluding the worst performers. Let B denote the number of best groups on placebo, as well as on active drug (across different doses), kept for the first stage analysis. Let γ denote the proportion of patients required to have the primary endpoint assessed in each subgroup of patients for the first stage analysis. After the first stage analysis, the majority of placebo-treated patients are recycled to be treated with active doses. Two best doses are identified by descriptive statistics, e.g. mean of utility, for the second stage, where utility is an overall quantitative representation of efficacy, safety, and tolerability (see Ref. [2]). In the end superiority is tested with each of the two active dose(s) versus placebo. Let μ_a,poc_, μ_p,poc_ be the mean for pooled active doses and placebo at POC stage, and μ_a,df_, μ_p,df_ the mean of the optimal dose and placebo at DF stage, then the tests for POC and DF are as follows.

(1) H_10_: μ_a,poc_ - μ_p,poc_ ≤ 0 vs. H_1a_: μ_a,poc_ - μ_p,poc_ > 0

(2) H_20_: μ_a,df_ - μ_p,df_ ≤ 0 vs. H_2a_: μ_a,df_ - μ_p,df_ > 0

**Fig. 1:**
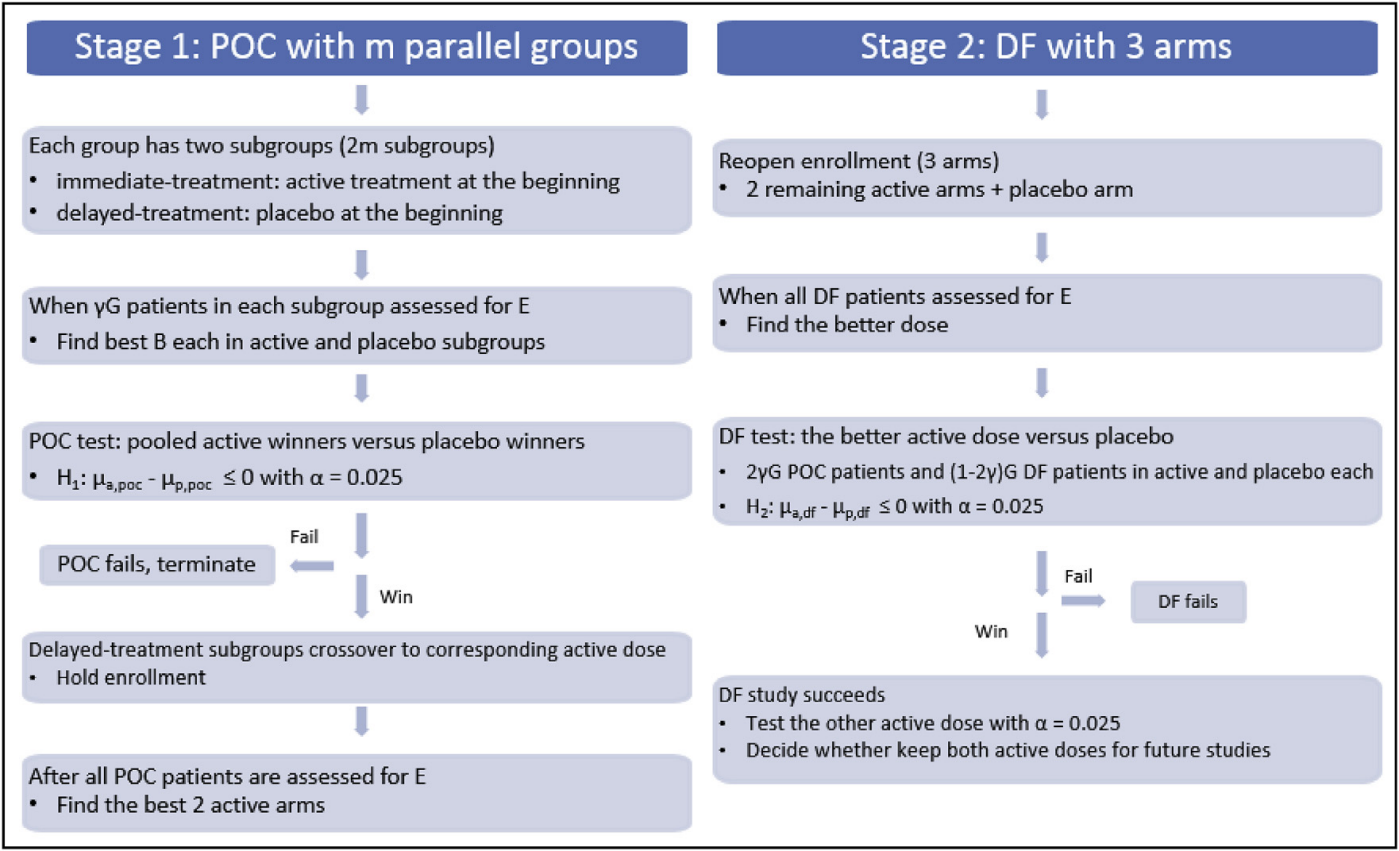
Study process of the adaptive design. The parameter G, per arm sample size adopted by a conventional DF study, is the maximum number of patients that can be enrolled in a group in the adaptive design. Conditions for other parameters are m ≥ 6, 0 < γ < 0.5, and 3 ≤ B ≤ m - 2.

Fig. 1 shows the study process of the adaptive design. We use G to denote the per arm sample size of a conventional DF study, and assume the conventional POC study per arm sample size to be 0.8G which is the same size for the POC test in the suggested adaptive design. Let us consider a conventional design with a POC study of 1 active arm and 1 placebo arm, and a DF study with 3 active arms and 1 placebo arm. For an example with m = 6, B = 4, γ = 0.2, the sample size is 4.2G for the suggested adaptive design and 5.6G for the conventional design.

**Fig. 2:**
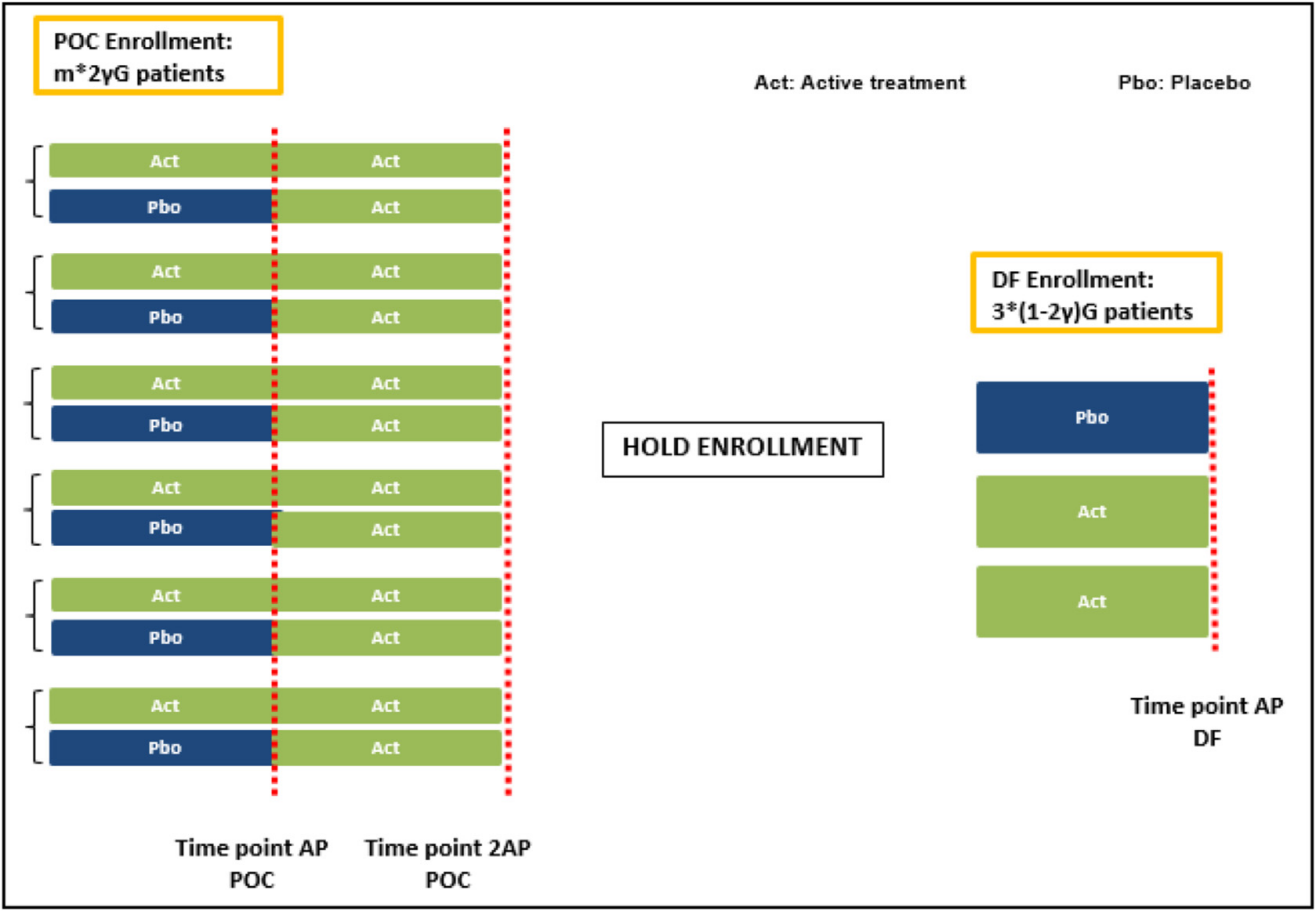
Enrollment and treatment flowchart. Patients are randomized into the POC per treatment sequences. There are 2 sequences for every dose: one sequence called the immediate-treated subgroup in which patients are treated with an active dose in both AP's; and the other sequence called delayed-treated subgroup in which patients are treated with placebo in the first AP and an active dose in the second AP. The randomization is 1:1 between the two sequences of a dose, and all doses are balanced in the POC enrollment. There may be a second enrollment, the DF enrollment, which does not start until the POC succeeds and the DF doses have been decided with the data from the POC stage.

Fig. 2 shows the enrollment and treatment flowchart. The POC enrollment randomizes γG patients to each of 2 m treatment sequences, where m sequences each consist of two AP's of active treatment at different dose levels, and the other m sequences each start with one AP of placebo followed by another AP of active treatment at different dose levels. Every two sequences that share the same active dose level form a dose group. After m*2γG patients have entered the study, the enrollment is held until the two DF doses are decided. Then the DF enrollment randomizes (1-2γ)G patients to each of a placebo arm and two active doses, and every patients are treated for one AP. Sometimes, an open-label active treatment period may be added after the AP to improve patients' willingness to be enrolled.

**Fig. 3:**
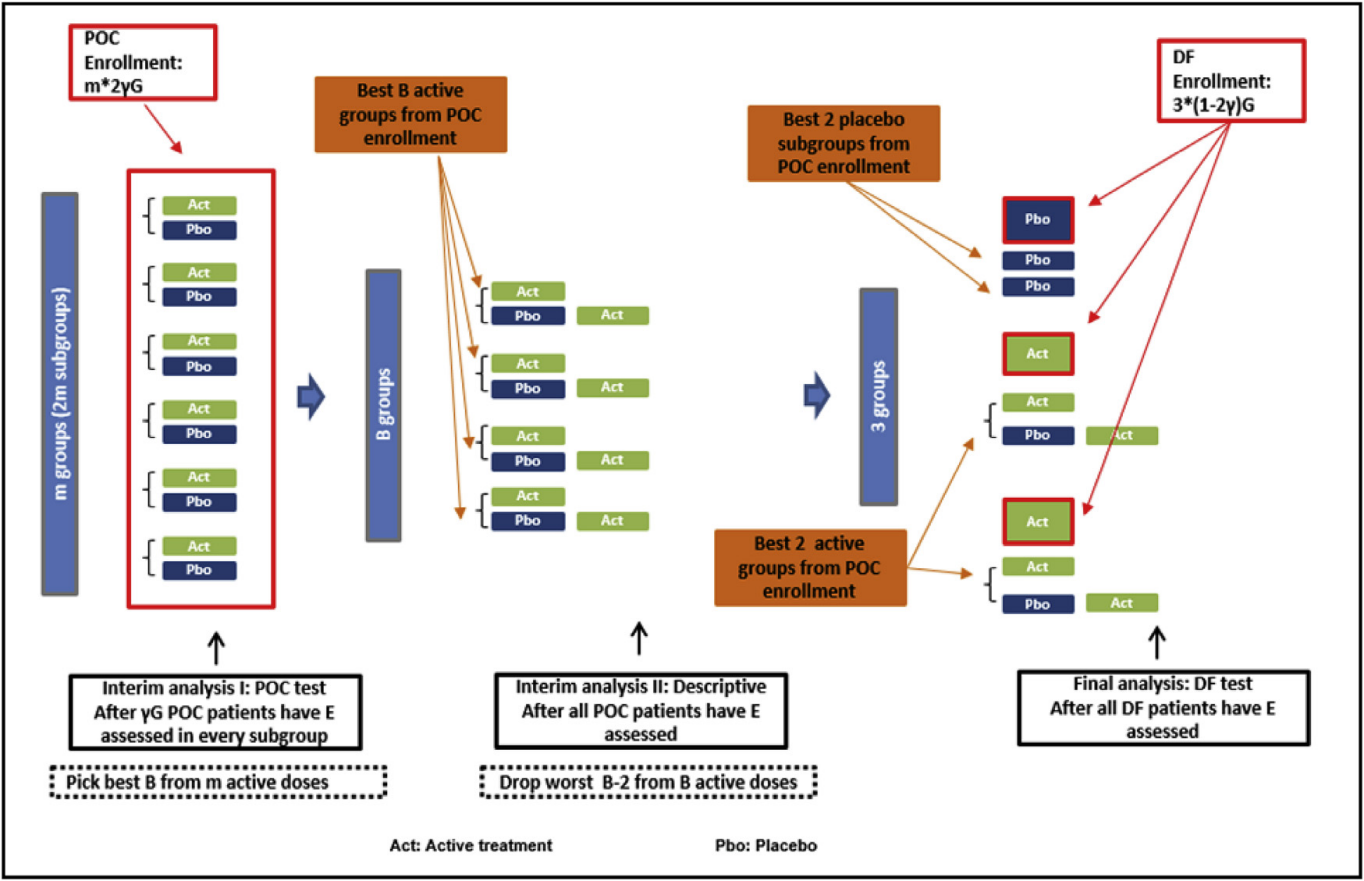
Statistical Analyses of Primary Endpoint. The first interim analysis is performed after γG (0< γ < 0.5) patients in each subgroup are assessed for E, testing superiority of the pooled active doses over placebo, with the better-performing B active subgroups pooled together (γBG patients) versus the better-performing B placebo subgroups pooled together (γBG patients). The second interim analysis is performed after all POC patients have E assessed, using descriptive statistics among the B better performing active doses, to select two doses for the DF enrollment. The final test is performed after all DF patients have E assessed, testing superiority of each of the best two doses over placebo, where the active group consists of patients from both the DF and the POC on the same dose and the placebo group consists of patients from the DF and the best two subgroups from POC.

Fig. 3 shows the statistical analyses of the primary endpoint. After γG (0< γ < 0.5) patients in each subgroup are assessed for E, find the best B subgroups among the actively-treated, and the best B subgroups among the placebo-treated. The best performers are identified by comparing descriptive summary statistics, e.g. mean value, and no inferential analysis is performed. With the better-performing B active subgroups (immediate-treated subgroups) and B placebo subgroups (delayed-treated subgroups), test superiority of the pooled active doses over placebo, i.e., conduct the superiority test (i) between the immediate-treated patients pooled together (γBG patients) and the delayed-treated patients pooled together (γBG patients) in the better-performing B active subgroups and B placebo subgroups. After all POC patients have been assessed for E, pick the best 2 dose levels, again by comparing descriptive summary statistics. After all DF patients are assessed for E, conduct the final test (ii) to compare each active dose to placebo in the quantitative order, i.e. first test the dose with a better descriptive statistic, where the patients on active treatment with the same dose from both POC and DF stage are pooled together, and the patients on placebo from the DF stage are pooled with the best 2 subgroups on placebo in the POC stage.

**Fig. 4:**
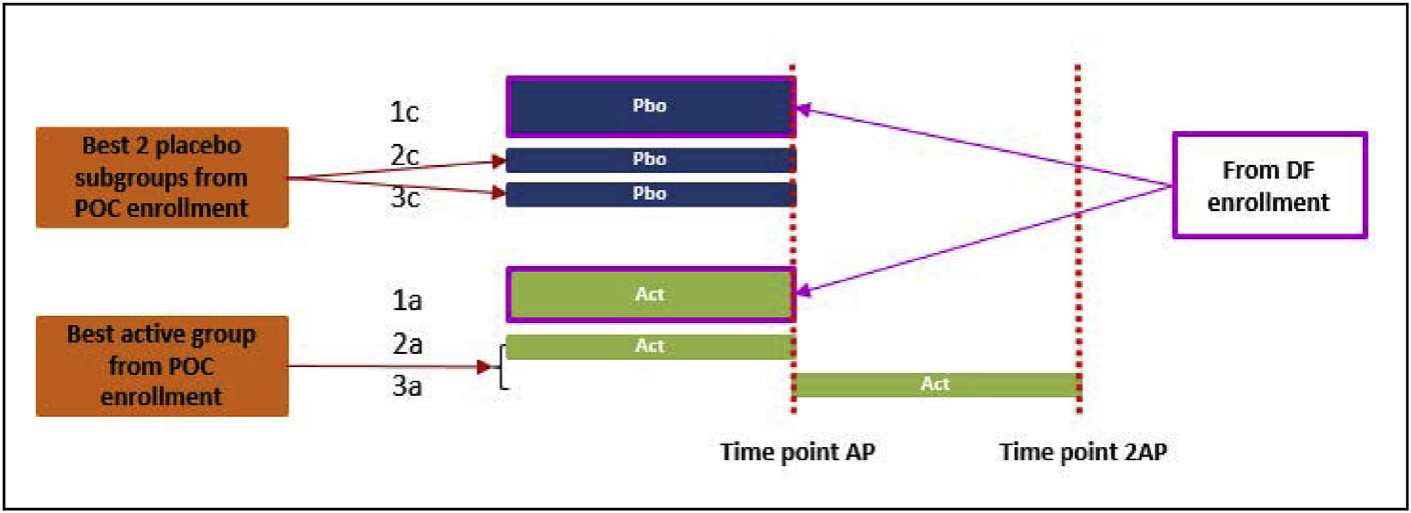
Patient data used for the first DF test. The active arm consists of the first AP data from (1-2γ)G DF patients (1a), the first AP data from γG POC patients (2a), and the second AP data from another γG POC patients (3a); the placebo arm consists of the first AP data from (1-2γ)G DF patients (1c), γG POC patients which form the best subgroup (2c), and another γG POC patients which form the second best subgroup (3c).

Fig. 4 shows the patient data used in the first DF test. In this primary analysis, the active arm consists of the first AP data from (1-2γ)G DF patients (1a), the first AP data from γG POC patients (2a), and the second AP data from another γG POC patients (3a); the placebo arm consists of the first AP data from (1-2γ)G DF patients (1c), γG POC patients which form the best subgroup (2c), and another γG POC patients which form the second best subgroup (3c). Obviously, (1a) vs (1c) is a fair comparison, (2a) vs (2c) is also a fair comparison. If there is no placebo effect then (3a) vs (3c) is also a fair comparison. If, however, placebo effect exists, after first AP of placebo treatment patients' status improves, then there is less room for improvement by the second AP of active treatment. Therefore it is a conservative estimate of the drug effect.

In addition to the above primary analysis, a sensitivity analysis can also be performed, where the active arm data only keeps (1a) and (2a), because (3a) may be impacted by placebo effect, period effect, or correlation with the placebo data; and the placebo arm data only keeps (1c) and (3c) if the placebo data of the patients in (3a) happen to be (2c), or (1c) and (2c) otherwise.

## 4 Comparison to conventional designs

### 4.1 Statistical operating characteristics

Based on the example introduced at the beginning of the article, we compare the proposed adaptive design to conventional design, using the concept of utility introduced by Ref. [2]. Because utility is a comprehensive assessment of efficacy, safety and tolerability, we assume that power based on utility to be 95% of the nominal power based on just the primary (efficacy) endpoint in the comparison between the same active treatment dose to placebo.

Since the POC and the DF share the same primary endpoint, we just use the DF portion of the 8 utility models introduced by Ref. [2]; see Table 1. Let utility of 100 represent the optimal dose and 15 the placebo. We assume the power based on utility for the comparison between the optimal dose and placebo to be 76% = 95%*80% when each group has 60 patients, (and 66.5% = 95%*70% when each group has 48 patients), which corresponds to a common standard deviation of 173 at a two-sided 0.05 alpha level.

**Table 1:** Different utility models considered in simulations.

Model	Utility distribution of active doses	Utility of placebo
1	82, 93, 100, 92, 81, 65	15
2	82, 93, 100, 85, 66, 45	15
3	65, 81, 92, 100, 93, 82	15
4	45, 70, 85, 100, 93, 82	15
5	86, 93, 100, 95, 90, 85	15
6	65, 80, 94, 100, 84, 66	15
7	45, 70, 85, 100, 75, 48	15
8	90, 95, 100, 97, 94, 91	15

Similar to [2]; two testing procedures are considered for the conventional DF study: one is to test the hypothesis for each dose the same way as if there were only one dose, and the other is to test these doses in a pre-specified sequence, e.g. from the high dose to the low dose and lower doses is tested only when the null hypothesis has been rejected for higher ones. The R package gMCP is used to assess the operating characteristics for the conventional approaches, see Ref. [1]. Assuming the conventional approach randomly picks 3 out of the 6 doses to conduct the DF study, gMCP is used to calculate the following operating characteristics:

(1) Power: the probability that the highest-utility dose (i.e. the optimal dose) is included in the DF study and its superiority over placebo is established, under the condition that the utility of the test drug is greater than placebo (at all dose levels).
(2) Type I error rate: the probability that superiority over placebo is established for at least one dose, under the condition that the utility of the test drug is the same as the placebo (at all dose levels).

With the conventional approach, DF power is a conditional probability conditioned on a successful POC study. Therefore, the power of winning both is the product of the POC power and the DF power. Under Model 1 utility distribution, (ignoring the alpha inflation with Strategy 1), the power for the conventional approach is in the range of 0.15–0.23 for winning both POC and DF.

As the power and the type I error rate are the worst when the results of different doses are independent [2], for the proposed adaptive design, simulations are performed under the situation where the results of different doses are independent. Among the patients that contribute to the active treatment of the final DF test, 20% also contribute to the POC test for the active treatment. Likewise, among the patients that contribute to placebo of the final DF test, 20% also contribute to the POC test for the placebo. The simulations account for this overlap.

In addition, there could also be patients who contribute to placebo in the POC test but to active treatment in the DF test. We account for this crossover correlation. If placebo effect exists, after first AP of placebo treatment patients' status improves then there is less room for improvement by the second AP of active treatment. Therefore, the crossover correlation is likely to be negative. For each level of the crossover correlation within the set of (−0.8, −0.6, −0.4, −0.2, 0, 0.2, 0.4), 10,000 simulation runs are performed to calculate the following operating characteristics:

(1) Power: under the condition that the utility of the test drug is greater than the placebo (at all dose levels), the proportion of successes with superiority established for both POC and DF, and the optimal dose is in the two doses picked for phase III
(2) Type I error rate: under the condition that the utility of the test drug is the same as the placebo (at all dose levels), the proportion of successes with superiority established for both POC and DF

With the Model 1 utility distribution, the results are shown in Table 2. The impact of correlation is negligible. (The situation is similar with other models.)

**Table 2:** Power and type I error rate for adaptive design.

Crossover correlation	Power	Type I error rate
−0.8	0.23	0.002
−0.6	0.22	0.002
−0.4	0.23	0.002
−0.2	0.23	0.002
0	0.22	0.001
0.2	0.23	0.002
0.4	0.22	0.001

The type I error rate is well controlled for the proposed adaptive design. The power is in the range of 0.22–0.23 for winning both POC and DF and the two doses selected for phase III include the optimal dose.

Under different scenarios of the utility model, the power for the proposed adaptive design and the conventional design is shown in Table 3, where power is defined as follows:

• with the conventional design the probability of (1) a successful POC and (2) the highest-utility dose is included in the DF study and its superiority over placebo is established, under the condition that the utility of the test drug is greater than the placebo.
• with the adaptive design the probability that superiority is established for both POC and DF, and the optimal dose is in the two doses picked for phase III, under the condition that the utility of the test drug is greater than the placebo.

**Table 3:** Power of adaptive design and conventional design.

Model	Conventional design	Adaptive design
1	0.15–0.23	0.22–0.23
2	0.11–0.20	0.21–0.22
3	0.17–0.23	0.22–0.23
4	0.15–0.20	0.21–0.22
5	0.19–0.24	0.22–0.23
6	0.15–0.23	0.22–0.23
7	0.11–0.20	0.19–0.20
8	0.20–0.24	0.22–0.23

In all the 8 models, the proposed adaptive design has advantage over the conventional design in terms of power. Specifically, in models where doses have relatively larger difference in utilities (Models 2, 4, 7), the advantage is the most pronounced, while in models where doses have similar utilities (Models 5, 8) the advantage is smaller. Other design elements of the conventional approach and the proposed adaptive design are displayed in Table 4. The results from Table 4, Table 5 have shown that the proposed adaptive design has the following advantages compared to a conventional design:

• Tests more active doses
• Higher power
• Smaller sample size
• Shorter overall study duration for POC and DF

**Table 4:** Design elements of adaptive design and conventional design.

Design elements	Conventional design	Adaptive design
Total number of patients	336	252
Number of active doses	4 or 3^a^	6
Patient years of exposure to placebo	24.8	24.8
Gap between POC and DF	Yes	No

a If POC dose is also one in the DF.

### 4.2 Clinical operations

For the example introduced in Section 3, with the conventional design, let us assume a maximum of 12 sites will be used for the POC study with a maximum monthly accrual rate of 12 patients and a 6-month linear ramp up, and 18 sites for the DF study with a maximum monthly accrual rate of 18 patients and a 4-month linear ramp up. We assume a 6-month gap between POC and DF for upper management communication/decision and possible agency consulting. The recruitment for the POC study takes about 12 months, followed by a 3-month blind follow up and a 1.5-month open-label follow up. The recruitment for the DF study takes about 15 months, followed by a 3-month blind follow up and a 1.5-month open-label follow up. So the total duration from first patient in (FPI) of POC and last patient out (LPO) of DF is about 42 months.

For the adaptive design, let us assume 15 sites will be used with a maximum monthly accrual rate of 15 patients and a 6-month linear ramp up. The recruitment will take about 20 months if ignore the enrollment hold between the POC and DF enrollments. The POC enrollment will be followed with a 6-month blind follow up, and the DF enrollment will be followed by a 3-month blind follow up and a 1.5-month open-label follow up. Let us the enrollment hold takes about 1.5 months. The total duration from FPI to LPO of the study will be about 32 months.

Based on Allergan's cost model with similar compound development, the estimated budget for the conventional design is $3.5 million for the POC and $7.5 million for the DF, and for the seamless adaptive design is $8.5 million, which is a 30% saving in funds.

Of course, there are some cons with the adaptive design as shown in Table 5. Mostly, adaptive designs demand more planning and upfront commitment. If the studied compound ends up with no efficacy, it will take a little longer for people to know with the adaptive design.

**Table 5:** Pros and Cons of adaptive design versus conventional design.

	Pros	Cons
Adaptive	• Less time, money, patients • More doses	• More upfront budget commitment and planning • Slower to know POC results (available after m*2γG patients reach AP)
Conventional	• Usual practice – more comfort • Quicker to know POC results (available after B*2γG patients reach AP)	• Only evaluating 3–4 doses vs 6 doses • More time, money, patients • Doubled effort in initiation of two studies, regulatory/ethics committee filings, documentation, etc.

## 5 Discussion

The authors propose an adaptive study design combining a phase IIa and a phase IIb study, where two stages share the same primary endpoint with the same assessment period, based on a previous proposed design in Ref. [2]. Compared to the previous design, the new design simplifies the study operation, hence makes it suitable for much more applications.

With the motivation example introduced at the beginning of the paper, the proposed adaptive design assesses 6 active doses using 252 patients without a gap between the POC and DF stage, while the conventional design assesses 3 or 4 active doses using 336 patients with a gap between the POC and DF stage. These differences translate into 30% saving in funds with Allergan's cost model. Other companies may gain different savings with different cost models, but the efficiency is obvious. Depending on the knowledge and marketing status of the class of the compound in development, omission of the gap between POC and DF may bring different values. In a relatively new drug class, a gap between POC and DF may be appreciated by the development team as well as the sponsor's senior management to fully communicate, discuss, and make decisions. If there has been good knowledge of the drug class and the path to market approval is clear, the omission of the gap can speed up the development.

## Appendix

A couple of important discussion points from Ref. [2] are repeated below.

### (A1) Rationale to have half patients in a delayed-treatment subgroup of every dose level at the beginning

Why not consider several active arms and one placebo arm in parallel with equal size? In the design proposed, a POC test is conducted at an early stage, where the active doses are combined excluding a couple of ‘losers’, and compared to the placebo group. To have a balance between the pooled active patients and placebo patients, 6 or 7 groups are used at the start of the POC, and each group is balanced between active and placebo (using a stratified randomization). After POC criteria have been met, only two delayed-treatment subgroups remain on placebo, and constitute the placebo group for the DF test later.

### (A2) Rationale to perform POC test with better-performing subgroups

If all active doses are included for POC test and ineffective active doses exist, the comparison power will be diluted. Therefore the worst active doses are excluded. The worst placebo subgroups are also excluded to mirror the algorithm for the active subgroups, otherwise the type I error may be inflated because better values are ‘picked’ for actively-treated patients.
